# Regenerative robotics

**DOI:** 10.1002/bdr2.1533

**Published:** 2019-06-11

**Authors:** Dana D. Damian

**Affiliations:** ^1^ Department of Automatic Control and Systems Engineering University of Sheffield Sheffield United Kingdom

**Keywords:** mechanotherapy, robotic implants, soft robots, tissue growth, tissue regeneration

## Abstract

Congenital diseases requiring reconstruction of parts of the gastrointestinal tract, skin, or bone are a challenge to alleviate especially in rapidly growing children. Novel technologies may be the answer. This article presents the state‐of‐art in regenerative robotic technologies, which are technologies that assist tissues and organs to regenerate using sensing and mechanotherapeutical capabilities. It addresses the challenges in the development of such technologies, among which are autonomy and fault‐tolerance for long‐term therapy as well as morphological conformations and compliance of such devices to adapt to gradual changes of the tissues in vivo. The potential as medical devices for delivering therapies for tissue growth and as tools for scientific exploration of regenerative mechanisms is also discussed.

## 1 TISSUE REPAIR

Tissue repair is a complex, long term, and physiologically demanding process requiring dynamic and optimal therapies (Eming, Wynn, & Martin, 2017). Patients suffering from conditions such as long‐gap esophageal atresia (LGEA), a congenital disease in which a section of the esophagus of 3 cm or more is missing, or short bowel syndrome (SBS), a devastating condition associated with massive loss or resection of the small intestine, skin burns, or bone deformities, are just a few examples of dramatic cases of tissue reconstruction that challenge patients and surgeons alike. Currently, the only effective treatment for LGEA consists of attaching sutures to the end of the esophageal ends, tying them off at the child's back and tightening them daily for weeks to encourage the tissue to elongate. During this treatment, the baby is sedated, and assessed with X‐rays (Foker, Kendall Krosch, Catton, Munro, & Khan, 2009). In SBS, because the child's remaining bowel length is insufficient to absorb nutrients and maintain health and growth, the treatments often target the dilation of the organ. Yet, the child is dependent on parenteral nutrition (PN, i.e., intravenous feeding) for months to years (Spencer et al., 2008) which can lead to morbidities like bloodstream infections and liver disease. These heroic surgeries performed by a few world experts are sadly primitive and morbid.

## 2 MECHANOTHERAPY

For a long time now, mechanotherapy—a form of physiotherapy using mechanical equipment to manipulate parts of the body, along with other exercises, massage, and so forth—has been recognized as effective for tissue repair, with treatments spanning months or years. Recent studies show that tissues grow in response to stimulative strain (mechanostimulation; Cezar et al., 2016; Folkman & Moscona, 1978)—this is nowhere seen more readily than in the growing child, in adults where exercise develops muscle mass and in pregnant women where skin expands to accommodate the growing fetus. It has been shown in vitro that this stimulation applied to cells can change their developmental trajectory toward death (necrosis), proliferation or differentiation (Folkman & Moscona, 1978). Clinically, the principle has been applied to induce bone growth, for skin grafts (Chua et al., 2016), wound healing (Huang, Holfeld, Schaden, Orgill, & Ogawa, 2013), growth of arteries (Kim et al., 2012) and esophagus expansion and elongation (Foker et al., 2009). However, the treatments rely on the surgeon's on‐site tactile perception or visual assessment, and empirical training, and thus are inconsistent. When much of the information about diseases is derived from advanced imaging technology (e.g., X‐rays, ultrasound) the importance of real‐time interaction with tissue in understanding immediate and long‐term effects of a therapy seems to be overlooked. With the knowledge of complex living tissues being at an early stage, inquiries on the optimal regimens of force application for tissue growth are needed. Presently, there are no studies about how tissue regeneration unfolds or is controlled during long‐term mechanostimulation. An in vivo device that enables informed and automated therapy would thus be extremely useful.

## 3 MEDICAL TECHNOLOGIES FOR TISSUE REPAIR AND GROWTH

Surgery‐assistive robotic devices have been shown to produce consistent outcomes in tissue repair, albeit their use, as a tool for surgeons (Shademan et al., 2016), is limited to manually‐operated, daily interventions (Sajadi & Goldman, 2015). Medical implants, such as the pacemaker, usually operate according to preprogrammed regimens (Copeland et al., 2004). Tissue engineering, on another front, uses engineered biomaterials (scaffolds) and growth factors to encourage host or donor cells to proliferate and grow new tissue. Despite successes in this field, challenges of cell death before vascularization of the scaffold, and patient‐specific factors affecting the tissue remodeling potential remain (Atala, Kasper, & Mikos, 2012).

## 4 SOFT ROBOTICS

Soft robots take advantage of both soft material engineering and robotic control to mimic natural properties, such as viscoelasticity, smooth motion, deformations, and self‐healing (Rus & Tolley, 2015). Soft robotic growth has also been investigated by a few studies for even search and rescue (Hawkes, Blumenschein, Greer, & Okamura, 2017; Rieffel & Smith, 2014; Sadeghi, Tonazzini, Popova, & Mazzolai, 2014), outside of the medical context. Despite recent developments in soft medical robots, such as soft neuronal sensors tested in vivo for days, and heart sleeves assessed in acute animal studies (Roche et al., 2017; Xu et al., 2014), there is no robot that has operated in vivo long term. The possibility to combine chemical, physical and electronic properties in soft‐matter substrates brings unprecedented flexibility in medical device customization, complexity, and mechanical compatibility for in vivo environments.

## 5 REGENERATIVE ROBOTIC TECHNOLOGIES

**Figure 1:**
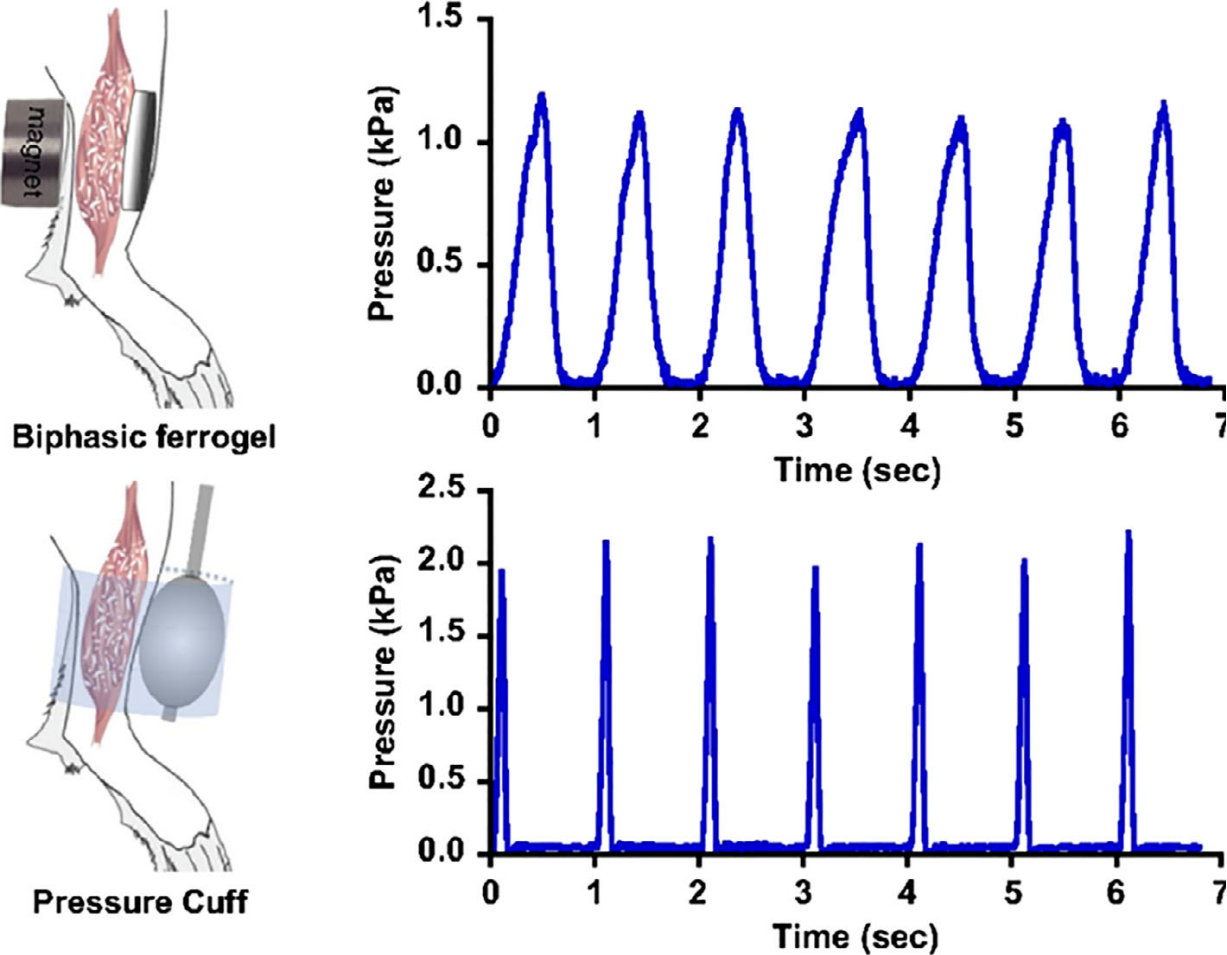
Biphasic ferrogels and pressure cuffs generate cyclic mechanical compressions. (Top) Schematic of biphasic ferrogel implant in mouse hind limb depicting orientation of ferrogel relative to skin, muscle tissue, and magnet (top left). Pressure profile of biphasic ferrogel undergoing repeated magnetic stimulations (top right). (Bottom) Schematic of pressure cuff on mouse hind limb depicting orientation of balloon and polycarbonate cuff relative to skin and muscle tissue (bottom left). Pressure profile of balloon cuff undergoing repeated inflations and deflations (bottom right; Cezar et al., 2016)

**Figure 2:**
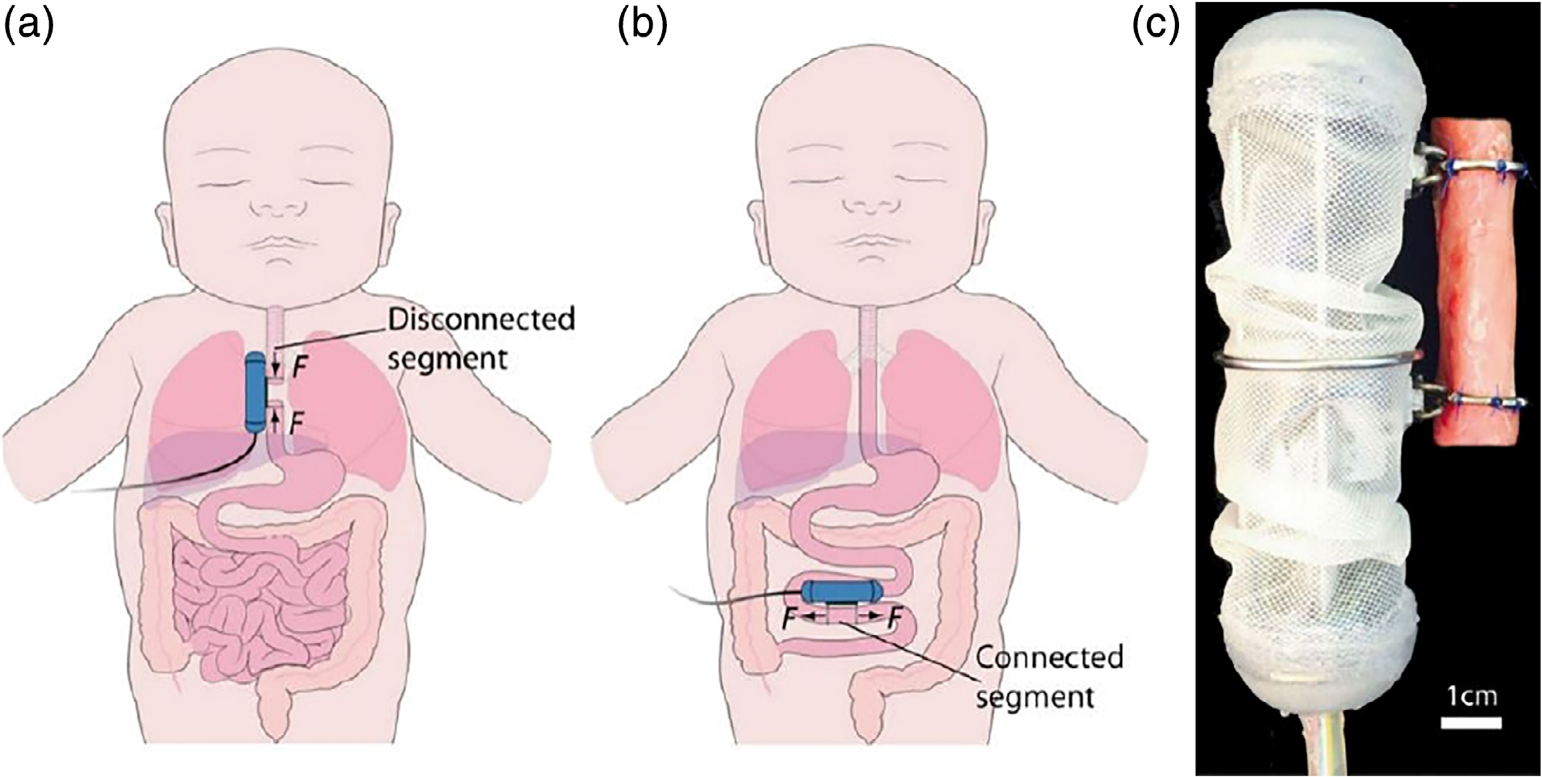
Robotic implant for tubular tissue growth. (a) For the treatment of long‐gap esophageal atresia, the implant applies forces (F) to disconnected esophageal segments. After inducing sufficient growth, the segments are surgically connected to form a complete esophagus. (b) As a potential treatment for SBS, the implant applies forces (F) to connected segment of bowel. By inducing sufficient lengthening to support the absorption of necessary calories and fluids, a dependence on intravenous feeding could be reduced or eliminated. (c) The robot is covered by biocompatible waterproof skin and is attached to tubular organ by two rings (esophageal segment shown). The upper ring is fixed to the robot body, whereas lower ring translates along the body (Damian et al., 2018)

Future tissue therapy should allow sustained, noninvasive, tissue‐responsive repair through autonomous, in‐situ, feedback‐controlled robotic implant technologies that regulate tissue growth by mechanostimulation (Damian et al., 2018; Miyashita et al., 2016). The realization of such devices would enable onboard clinical expertise and delivery of effective therapy at all times, as well as the acquisition of in vivo tissue data to research growth mechanisms, which is impossible in current clinical and research practice. This technology could customize treatments by exploiting natural growth capabilities of the remaining tissue. Novel regenerative technologies are emerging and combine the interdisciplinary knowledge from these fields in order to provide more reliable, controlled, on‐demand tissue therapies for wound healing and tissue growth. Two examples are given below:

1. Therapeutic hydrogel substrates: Cezar et al. developed an actuated biologic‐free ferrogel able to apply, under a magnetic field, mechanical compression to damaged skeletal muscle (Cezar et al., 2016). Their results showed that this mechanical intervention positively affected the host inflammatory response, by significantly reducing the fibrotic capsule around the gel after 2 weeks of implantation in mice hind limb. Furthermore, the cyclic application of the mechanical compression led to enhanced muscle regeneration compared to no‐treatment controls, indicating the potential of regenerative therapies through mechanotherapy. The hydrogel substrate allowed a profile of mechanical compression that produced a better outcome than the acute compression profile of inflating balloon cuffs around the mouse limb (Figure 1). In the reported work the mechanotherapy regimens were predefined, thus further envisaged improvement could be sensing incorporation to adapt these regimens to changes of the tissue in time, such as stiffness.
2. Robotic implants: Damian et al. developed robotic implants that show capabilities to regulate and enhance tissue growth through mechanostimulation: by applied forces to esophageal and bowel tissue in swine animals (Damian et al., 2014, 2018; Price, Machaidze, Jaksic, Jennings, & Dupont, 2016; Figure 2). Supported by advancement in mechatronic design allowing week‐long robot operation in vivo, an average of 77% of new esophageal tissue in 9 days was achieved, with 63% of lengthening due to muscle cell proliferation and 37% due to collagen formation (fibrosis). These studies have also revealed a full range of unrecognized challenges owing to the stiff and fixed implant design operating long‐term in a harsh in vivo environment. Due to the interaction between the rigid implant and tissue, we ascertained that the fibrosis level was notable. Also quick bursts of damaging forces on the tissue at points of contact with the robot generated by the host tissue's dynamics were difficult to counteract. Moreover, the robot was limited in the length of tissue it can grow due to the fixed mechatronics design.

## 6 CHALLENGES AND OPPORTUNITIES

While the aforementioned technologies, as currently demonstrated, obviate the need of cell culture and growth factors, the underlying techniques in tissue engineering may be combined and incorporated to augment outcomes. Advancing these technologies to the clinic will entail overcoming a conglomerate of technological challenges that are derived from stringent clinical and biological requirements, as outlined in Table 1.

**Table 1:** Clinical and technological challenges in developing regenerative technologies

Clinical challenge	Benefit	Technological challenge
Safe	Patient safety, reduction of inflammation	Bio‐ and mechanically‐compliant with tissues, controllable
Long‐term therapy	Personalized at‐all‐times treatment with monitoring and therapy delivery throughout the healing process	Programmability, autonomy, adaptive control, fault‐tolerance
Support tissue lengthening	Lengthen with the tissue	High and sustainable deformation
Minimally or noninvasive	Fast recovery time, patient comfort, reduce inflammation	Miniaturization, biodegradability

One requirement to create safe implantable technologies for patients is to develop those devices that are biocompatible and mechanically compliant with the tissue. It has been demonstrated that the latter feature reduces the inflammatory response of the body (Moshayedi et al., 2014). Soft sensors, actuators, and robots are able to conform and comply with the geometry and mechanics of soft tissues, thus providing a significant potential to meet these requirements (Bartlett, Markvicka, & Majidi, 2016; Ilievski, Mazzeo, Shepherd, Chen, & Whitesides, 2011; Lum et al., 2016). Importantly, these technologies should also be controllable, enabling the possibility of human intervention in device operation to override autonomous device operation whenever needed. While current treatments provide short‐term and periodic interventions, long‐term therapies need to provide the possibility to intervene at any time during the healing and regenerative process, to maximize the physiological results and minimize the pathological factors. Endowing these devices with autonomy is key for such therapies, as such autonomous devices would have to have access to on‐site sensor readings and apply adaptive tissue‐responsive therapies. Autonomy has been demonstrated in surgical tasks using the Da Vinci robot, which is a robot comprising of multiple robotic arms designed to perform surgery inside the body through key‐hole incisions (Shademan et al., 2016). The autonomous surgery with the Da Vinci robot combined suturing tools and multimodal imaging, sensing and high‐resolution positioning for soft tissue surgery. The results of the autonomous robotic surgery were superior to manual surgery, laparoscopy, and robot‐assisted surgery for an intestinal anastomosis with ex vivo porcine tissues and living pigs. The implementation of autonomous implantable technologies requires accurate knowledge of the tissue for decision‐making and for safe, meaningful and effective therapeutic actions. Additionally, it is important that autonomy is extended to the resilience of the device; as an implantable technology residing in inaccessible places, the device must be able to operate at all times, isolating or compensating for potentially occurring internal device faults (Terryn, Brancart, Lefeber, Van Assche, & Vanderborght, 2017). Implants for tissue healing and regeneration also need to geometrically adapt to the lengthening tissue as a result of the artificially or biologically induced tissue growth. The latter one is especially important for pediatric patients. Current approaches consist of materials that change size due to biodegradability or due to elasticity (Feins et al., 2017; Perez Guagnelli et al., 2018). Lastly, it is desirable for such implantable technologies to be minimally invasive or low‐profile, which is critical for pediatric patients in particular. This requirement has been addressed using materials that can be deployed from small to large structures due to swelling or unfolding (Hu, Lum, Mastrangeli, & Sitti, 2018; Miyashita, Guitron, Li, & Rus, 2017).

These challenges are interdisciplinary and materialize into an engineering question of how to create a mechanically malleable robotic implant that is able to deform and induce tissue stimulation to effectively reconstruct and restore tissue performances with minimum human intervention. Apart from the clinical impact of the regenerative technologies, they also have the potential to shed light on scientific questions related to the mechanisms of growth and scar reduction at both tissue and cellular levels. How to optimize cell regeneration in a closed loop control? What are viable traction force regimens that lead to maximization of cell proliferation? What are the in silico and in vitro models that assist the in vivo tissue growth optimization and reduce animal trials and speed developments to clinical use? How to model and estimate tissue healing and inflammatory response with limited sensory information in an in vivo dynamic environment? Supported by rapid advancements in the fields of tissue engineering, biology and robotics, it is promising that these interdisciplinary questions will find an answer in the following years, thus providing the much‐needed treatment to patients young and old.
